# Jesus Practiced Advance Care Planning: Biblical Basis and Possible Applications

**DOI:** 10.1089/pmr.2020.0012

**Published:** 2020-10-22

**Authors:** Grace Johnston

**Affiliations:** School of Health Administration, Dalhousie University, Halifax, Nova Scotia, Canada.

**Keywords:** advance care planning, culture, religion

## Abstract

***Background:*** Persons from a Christian tradition may have concerns that impede advance care planning for end of life. Sharing how Jesus practiced advance care planning may provide a pivot point to help ameliorate this problem.

***Objective:*** To present a novel approach to advance care planning from a Christian tradition.

***Evolution of the Novel Approach:*** Experiential learning that resulted in the novel approach is described using Kolb's learning cycle: proceeding from concrete experience to reflective observation followed by abstract conceptualization and then active experimentation.

***Results:*** The novel approach builds on events toward the end of Jesus' life to demonstrate how he practiced advance care planning: telling those close to him that he was going to die even though they did not want to hear this, participating in a celebration of his life on Palm Sunday, sharing a Last Supper with those close to him, showing them how he wanted to be remembered, asking his friends to pray with him in the garden of Gethsemane, and saying to his mother that John would care for her. Questions related to these events are posed for use by health and spiritual care professionals to innovatively engage persons in advance care planning.

***Discussion:*** This approach might be adapted for persons of other religious traditions by exploring their sacred teachings. It is proffered for others to explore, adapt, and evaluate for its utility in initiating and facilitating advance care planning.

## Background

Advance care planning is a process that supports persons in understanding and sharing their values, life goals, and preferences regarding their future care.^1^ Spoken or unspoken, with conscious awareness or not, persons from a Christian tradition may have concerns that undermine advance care planning, including a conviction that discussion of dying portends death, belief in prayer for a cure^2,3^ so one must not give up,^2^ moral positions on prolonging life,^2^ and fear of judgement by the divine.^2,3^ Although Jesus did not issue specific teaching on advance care planning for end of life, his words and actions as his death approached may aid in community engagement by pivoting away from these concerns.

Although much has been written by theologians on death and dying,^4^ little has been written in recent decades about the religious dimensions of hospice-palliative care even though it is known to be important in practice.^5^ Many resources discuss advance care planning from a medical perspective,^6^ but few address the interface between advance care planning and religious traditions. This report begins to tackle this knowledge gap using events from the life of Jesus as an exemplar of how health and spiritual education providers might engage audiences in advance care planning using insights from a religious tradition.

“Religious tradition” is used rather than “religion” to encompass the many and ever-changing institutional forms of such traditions as well as individual experiences.^5^ Even as some nations transition into a post-Christian era, we remain affected by the legacy of Christian teachings. In particular, among those dying, many around the world retain a direct or indirect influence from Christian thought.

## Objective

The objective of this article is to present a novel approach to advance care planning from a Christian tradition. The goal is to encourage greater participation in advance care planning.

## Evolution of the Novel Approach

My experiential learning that resulted in this novel approach is described *post hoc* using Kolb's four-step learning cycle: proceeding from concrete experience to reflective observation followed by abstract conceptualization and then active experimentation.^7^ My experience is grounded in my palliative care and other research, my faith journey, and the approach of Dame Cicely Saunders.^5,8,9^ The preparation of this reflective article did not require an ethics board review.

### Concrete experience

A decade ago, a study on religious coping and the use of intensive life-prolonging care^2^ triggered my interest. It found that patients with advanced cancer who had a high level of religious coping (predominately Christian) were less likely to have an advance care plan and more likely to seek intensive life-prolonging care in their last week of life.^2^

### Reflective observations

My prior research on collaboration^10^ and interprofessional learning,^11^ and working with trainees (Parker,^12^ Maddalena,^13^ and Orton^14^) on a palliative approach to care within cultural groups with faith traditions formed a basis for further observation and reflection. I learned the value of bridging across differing mindsets to enable people to work together for a common purpose. From research on improving rates of Pap screening,^15^ I learned that endorsement by the local religious leader (e.g., priest, imam) was important to reach women who practiced their faith. I mused about the apparent divide today between Christian churches and the healthcare system in Canada.

This led me to explore what guided Saunders and her leadership in founding the global hospice movement, with particular attention to the role of her Anglo-Christian faith.^3^ It is now >50 years since Saunders began her hospice work. New Anglo-Christian leadership is emerging, including MacLaren on the great spiritual migration,^16^ Wright on the meaning of the resurrection of Jesus,^17^ and Rohr on the universal Christ.^18^ These authors quote Bible verses and events in the life of Jesus, as did Saunders,^5^ to provide guidance on addressing issues of the day.

### Abstract conceptualization

A novel approach that bridges across Christian and palliative care orientations might pivot audiences away from preconceived ideas that could undermine open discussion on preparing for one's death and dying. The adage “What would Jesus do?”^19^ offered the starting point that is presented in the following results.

### Active experimentation

My reflections on how Jesus practiced advance care planning had traction when speaking with more than a dozen Christian leaders and when invited to present to five different groups (two Baptist, one nondenominational, one Roman Catholic, and one that comprised cancer survivors) in Nova Scotia for the past five years. While discussing palliative care and advance care planning, I would briefly include my perspective on how Jesus practiced advance care planning. As I shared this novel perspective, I noticed that the listeners became more attentive, seemed to relate to what I was sharing, and lively discussion ensued. I did not instruct people on how to actually complete an advance directive. Rather, I referred them to professionals who worked in the health system in their geographic area, and when possible, came from their religious or group's tradition, to provide this education.

## Results

The novel approach to advance care planning is based on events in the last weeks of the life of Jesus. These events can provide a model for discussing one's pending death with loved ones, praying and following religious traditions. Jesus told those close to him that he was going to die even though they did not want to hear this, participated in celebrating his life on Palm Sunday, shared a Last Supper with those close to him, showed them how he wanted to be remembered, asked his friends to pray with him in the garden of Gethsemane, and said to his mother that John would care for her (see Table 1, column 1). The second column lists Biblical references that can be used for further exploration. The third column proffers a starting point for discussion on advance care planning with persons who value their Christian tradition by posing questions that flow directly from these events in the life of Jesus.

**Table 1.** Jesus Practiced Advance Care Planning and How This Might Engage Persons from a Christian Tradition

Jesus' words and actions	Biblical gospel reference	Possible applications
Spoke openly about his pending death despite the resistance and dismay of others.	Matthew 16:21–23; Mark 8:31–33	Are you willing to recognize, accept, and discuss your death? Why might this be important?
Participated in a celebration of his life on Palm Sunday five days before he died.	Matthew 21:1–11; Mark 11:1–11; Luke 19:28–40; John 12:12–19	Might you want to celebrate your life even as your death approaches?
Arranged an intimate Last Supper with his friends where he shared symbols of remembrance.	Matthew 26:17–29; Mark 14:12–25; Luke 22:7–30; John 13:18–30	With whom do you want to spend time? Any favorite meals? Eating places? Symbols to share?
Asked his friends to be with him while he prayed in the garden of Gethsemane.	Matthew 26:36–44; Mark 14:32–40; Luke 22:39–45; John 18:1	Might you want a quiet place? Place of prayer? Friends or prayer partners with you?
Planned his mother's care after he died by having John care for her.	John 19:26–27	Are there others for whom you are responsible? Who do you want to care for them?

The novel aspect of this approach is the bridging from events familiar to Christians to questions that may be asked in preparing an advance care plan. By juxtaposing what is accepted by Christians with what is familiar to advance care planning educators, a confluence and open space are generated for creative dialogue and reflection on advance care planning that otherwise is unlikely to occur.

## Discussion

The purpose of this article is to provide health and spiritual care professionals with a novel approach that might help them creatively engage with Christians in discussion around advance care planning. Kolb's learning cycle was used *post hoc* to describe the evolution of this novel approach that is grounded in my own experience, the life of Jesus, and an understanding of the approach of Saunders.^5,8,9^ The events in the life of Jesus cited in the results section of this article are typically well accepted by Christians; they occur during the holiest week in the Christian calendar.

Saunders saw the journey into death as one of mystery, love, acceptance, and spiritual growth for patients and staff.^8^ This is the antithesis of an ethos of fear and judgement. It is an acceptance of death as part of life, supported by community and prayer. She believed in the power of religion to make sense of suffering and foster a “good death.”^8,9^

Saunders took moral positions throughout her life. For example, she was opposed to euthanasia, but did not impose her views on others.^8^ She saw each person as walking their own spiritual journey fully supported by others^8^ and encouraged openness to all religious traditions.^5^

Saunders aimed for a balance between the wisdom of religion and the science of medicine.^5,9^ Although tensions and concerns arose about her boundary-blurring ways,^8^ she moved forward amid unanswered questions.^8^ She sought the wisdom of Christian leaders of her time^8^ while pragmatically improving hospice care through innovations grounded in research.^8^ Thus, the development of this report on how Jesus practiced advance care planning has parallels to the development of hospice care by Saunders.

Advance care planning includes an exploration of one's values.^1^ Some people are unclear or inconsistent in expressing their values.^20^ Thus, values assessment tools are emerging.^20^ However, statements on one's religious tradition are typically broad, for example, “What importance do religious/spiritual beliefs play in guiding your medical treatment?”^21^ Therefore, an approach for entering into deeper exploration is timely.

This novel approach is provided for others to explore, adapt, and evaluate in regard to initiating and facilitating advance care planning, and improving palliative supports and outcomes among persons interested in a Christian perspective. Christian views are not homogeneous and they appear to be in a state of flux,^16^ thus potentially opening up new opportunities for innovative thinking. Hopefully, this study will be a useful addition to the advance care planning toolkit and a basis for further research and discussion.

Table 1 is an icebreaker to engage a Christian audience in advance care planning. It does not provide depth or comprehensiveness in Christian theology. This report is only from a Christian perspective but hopefully is an exemplar for producing parallels in other faith traditions. The investigation of what is not covered in this report is left to others. This article presents a novel snapshot on advance care planning for persons from a Christian tradition.
